# Poly(1-trimethylsilyl-1-propyne)-Based Hybrid Membranes: Effects of Various Nanofillers and Feed Gas Humidity on CO_2_ Permeation

**DOI:** 10.3390/membranes8030076

**Published:** 2018-09-05

**Authors:** Zhongde Dai, Vilde Løining, Jing Deng, Luca Ansaloni, Liyuan Deng

**Affiliations:** Department of Chemical Engineering, Norwegian University of Science and Technology, 7491 Trondheim, Norway; zhongde.dai@ntnu.no (Z.D.); vilde.sl@gmail.com (V.L.); jing.deng@ntnu.no (J.D.); luca.ansaloni@ntnu.no (L.A.)

**Keywords:** CO_2_ separation, hybrid membranes, high free volume polymers, PTMSP, ZIF

## Abstract

Poly(1-trimethylsilyl-1-propyne) (PTMSP) is a high free volume polymer with exceptionally high gas permeation rate but the serious aging problem and low selectivity have limited its application as CO_2_ separation membrane material. Incorporating inorganic nanoparticles in polymeric membranes has been a common approach to improve the separation performance of membranes, which has also been used in PTMSP based membrane but mostly with respect to tackling the aging issues. Aiming at increasing the CO_2_ selectivity, in this work, hybrid membranes containing four types of selected nanofillers (from 0 to 3D) were fabricated using PTMSP as the polymer matrix. The effects of the various types of nanofillers on the CO_2_ separation performance of the resultant membranes were systematically investigated in humid conditions. The thermal, chemical and morphologic properties of the hybrid membranes were characterized using TGA, FTIR and SEM. The gas permeation properties of the hybrid membranes were evaluated using mixed gas permeation test with the presence of water vapour to simulate the flue gas conditions. Experiments show that the addition of different fillers results in significantly different separation performances; The addition of ZIF-L porous 2D filler improves the CO_2_/N_2_ selectivity at the expenses of CO_2_ permeability, while the addition of TiO_2_, ZIF-7 and ZIF-8 increases the CO_2_ permeability but the CO_2_/N_2_ selectivity decreases.

## 1. Introduction

Since the late 1960s, membrane-based separation processes have gradually been recognized as feasible alternatives to conventional purification and separation processes [1]. Compared to conventional separation technologies, membrane separation has advantages including small footprint, high energy-efficiency as well as safe, environmentally friendly and easy operation. Membranes have been applied in a wide range of gas separation applications, such as natural sweetening (acid gas removal), biogas upgrading, hydrogen recovery, N_2_ production and organic vapour recovery [2]. Nowadays, using membranes for CO_2_ capture from flue gas (post-combustion) or syngas (pre-combustion) has attracted much attention [3]. Various types of membrane materials and membrane processes have been developed for CO_2_ capture, including polymeric membranes, inorganic membranes and hybrid membranes. Among them, polymeric membranes are the most commonly used and intensively studied owing to its good processability and relatively low cost.

Polymer membranes usually separate gases by preferential permeation under a pressure gradient following the solution-diffusion mechanism. However, fast permeation through a polymeric membrane is usually achieved by compromising selectivity: The permeability and selectivity are subjected to a trade-off as being described by the Robeson upper bound [4]. Developing new membranes with both high permeability and selectivity has thus been a major research goal. Attempts to overcome the performance limitation are generally based on designing and controlling the materials chemistry or nanostructure of the membranes to create more preferential sorption and/or diffusion of the target penetrants. One promising strategy is to synthesize multicomponent hybrid membranes, in which a secondary component (usually inorganic nanoparticles) is introduced and evenly embedded into the polymer matrix. The additives can be porous or non-porous nanoparticles (0 to 3D) [5] and/or liquid plasticizers [6,7,8]. In the past few years, various nano- or micro-porous particles have been applied in fabricating hybrid membranes, including zeolite, carbon molecular sieve, different metal organic frameworks (MOFs) and covalent organic frameworks (COFs). Non-porous particles include metal oxides, silica, carbon nanotubes and graphenes. Graphene oxide are also used to fabricate hybrid membranes [5,9]. Generally, due to the non-ideal interface between the additives and the polymer phase, adding nanofillers into polymeric matrix of a membrane will result in significant improvement in gas permeability but relatively moderate enhancement in gas selectivity [10]. 

PTMSP is one of the representative high free volume polymer possessing the highest CO_2_ permeability among dense polymeric membranes. The high gas permeability is attributed to the exceptionally high free volume of the polymer [11]. The large and interconnected domains within the polymer matrix allows a rapid diffusion of gas, leading to the high gas permeability across the membrane [11]. The CO_2_ permeability is reported to be in the range of 18,000–5200 bar [12,13,14]. However, the CO_2_/N_2_ selectivity remains relatively low (<5) [12]. According to process simulation, for membranes with a sufficient selectivity, high gas permeability is a more important property to consider from the economic point of view [15]. The poor selectivity has limited its industrial application. Increasing the selective features of this material is thus of primary importance to boost its practical use in CO_2_ separation. Adding inorganic nanofillers into polymeric matrix has been proven to be an effective way of improving the gas separation properties [16,17]. 

Zeolitic imidazolate frameworks (ZIFs) are a family of the metal organic frameworks (MOFs) usually with zinc or cobalt as metal nodes and imidazolate (or imidazolate derivative) as organic linkers [18]. ZIFs have been extensively used in hybrid membrane fabrications as porous nanoparticles. Among the series of ZIFs, ZIF-8 is one of the most widely studied materials with various applications in gas storage, catalysis and gas separation [19]. Many researchers have reported that adding ZIF-8 into polymeric membranes could not only improve the gas permeability but also enhance selectivity [20,21,22]. ZIF-7 is also well studied as nanofillers in different polymeric membranes. It is found that ZIF-7 nanoparticles could effectively improve both the CO_2_ permeability and CO_2_ selectivity over other gases (e.g., N_2_ and CH_4_) [23,24,25]. ZIF-L, a two-dimensional zeolitic imidazolate framework, is relatively new, which was firstly developed by Wang et al in 2013 [26,27]. Compared to the conventional ZIF particles, ZIF-L has a distinctive leaf-shaped morphology and cushion-shaped cavity with a dimension of 9.4 Å × 7.0 Å × 5.3 Å between layers, which is well suited to accommodate CO_2_ molecules [28]. In addition, the intrinsic high-aspect-ratio of the 2D particles is preferable for improving gas selectivity [29]. It has been used to fabricate hybrid membranes with different polymers for gas separation and pervaporation [30]. However, the comparison of the effect of different ZIFs on the CO_2_ transport properties of a given polymeric matrix, especially the high gas permeable matrix, is still not completely disclosed.

Non-porous nanofillers have also been reported to be able to significantly change gas separation performances, typically due to specific affinity with a target molecule (transport via selective surface diffusion phenomena) or interactions with polymeric chain packing, leading to higher free volume within the polymer matrix [31]. Various non-porous additives has been used to fabricate hybrid membranes for gas separation purpose, such as TiO_2_, fumed silica and other metal oxides [5]. Due to the characteristic properties such as inexpensive, high hydrophilicity as well as good chemical and thermal stabilities [32,33], TiO_2_ has been included as the non-porous nanofiller for comparison with the ZIFs in this study.

Furthermore, as in real gas streams flue gas is always saturated with water vapor and in many cases water vapor is found to strongly influence the properties of the membranes, it is critical to investigate the effects of humidity in the feed gas on the separation performance of the membranes. To the best of our knowledge, the influence of water vapor in separation system on the permeation properties of PTMSP-based hybrid membranes has never be reported.

In the present work, four different nanoparticles were employed as additives to prepare PTMSP-based hybrid membranes, including three porous ZIF nanoparticles (ZIF-8, ZIF-7 and ZIF-L) and one non-porous nanofiller (TiO_2_). The resultant hybrid membranes were systematically investigated using various techniques, including TGA, FTIR and SEM and mixed gas permeation tests with controlled humidity (0–100% relative humidity). The separation performance data are analyzed and the influence of the various fillers on the gas transport mechanism through the hybrid membranes is discussed.

## 2. Experimental

### 2.1. Materials

**Figure 1:**
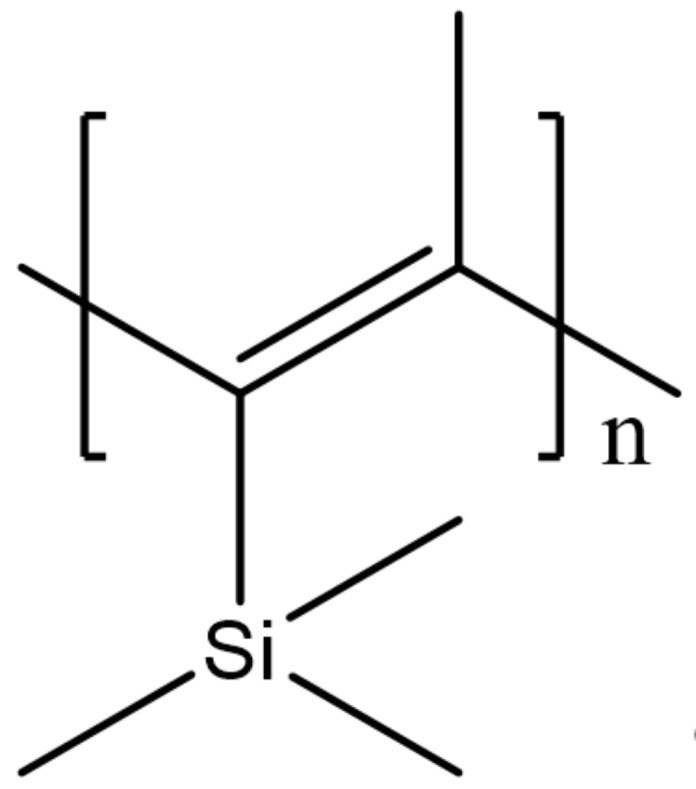
Chemical structure of poly(1-trimethylsilyl-1-propyne) (PTMSP).

**Figure 2:**
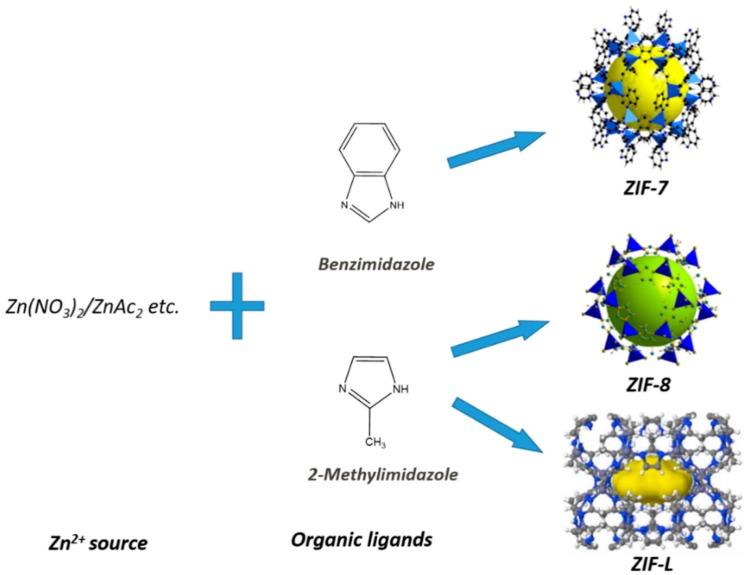
Structure and preparation route of ZIF-7, ZIF-8 and ZIF-L [26].

PTMSP was supplied by Fluorochem, Morrisville, PA, USA (chemical structure shown in Figure 1). ZIF-8 nanoparticles (trade name of Basolite^®^ Z1200, solid state, Sigma-Aldrich, Steinheim, Germany), chloroform and n-haxane were purchased from Sigma-Aldrich, Steinheim, Germany. ZIF-7 and ZIF-L were synthesized following the procedure described by Zhong et al. [26] and Li et al. [23], respectively. The structure and common preparation route for ZIF-8, ZIF-L and ZIF-7 are given in Figure 2. TiO_2_ nanoparticles were kindly supplied by SINTEF Industry, Oslo, Norway. Before use, all the particles were dried in a vacuum oven at 60 °C overnight to ensure the complete removal of moisture. The mixed gases used for permeation testing contains 90 vol% N_2_ and 10 vol% CO_2_. 99.999 vol% CH_4_ was used as sweep gas. All the gases were purchased from AGA, Trondheim, Norway. 

### 2.2. Membrane Preparation

Self-standing PTMSP membranes of approximately 50–100 μm were fabricated by casting the polymer/additive mixture on a glass plate using a casting-knife. The polymer/additive blend were prepared as described by Zhang et al. [34]. In brief, solution A (nanoparticles in either chloroform or n-haxane) was sonicated for 4–5 min using a Sonics Vibra-CellTM VC 70T (Leuven, Belgium) and then mixed into solution B (a highly viscous solution of PTMSP in a small amount of solvent). The mixture of A and B was then sonicated again for 4 × 45 min before the solution was cast onto a glass plate. The solution on the glass plate was covered by a glass container to slow down the solvent evaporation. 

The content of nanofillers added into the membrane matrix was determined using Equation (1).

(1) $$\Omega_{ad}=\frac{W_{ad}}{W_{ad}+W_{PTMSP}}*100\;$$

where $W_{ad}$ and $W_{PTMSP}$ are the mass of additives and PTMSP polymer, respectively. 

### 2.3. Membrane Characterization

A TG F1 Libra (NETZSCH, Selb, Germany) was used to perform a thermo-gravimetric analysis (TGA) of the hybrid membranes. Around 10 mg of sample were used to perform the test. The samples were analysed in the temperature range from room temperature (RT) to 700 °C at a constant heating rate of 10 °C min^−1^ with N_2_ atmosphere.

Fourier Transform Infrared Spectroscopy (FT-IR, NicoletTM iSTM 50, Thermo Fisher, Oslo, Norway) was used to determine the chemical structures of all the components in the hybrid membranes. The wavelengths employed were in the range of 650–4000 cm^−1^ and the given spectra is an average of 16 scans. 

Scanning electron microscope (SEM, TM3030Plus, Hitachi, Espoo, Finland) was used to investigate the morphology of the membranes. Both cross section and surface samples were sputter coated (Q150R Rotary-Pumped Sputter Coater/Carbon Coater, Quorum, Laughton, East Sussex, UK) with a thin gold layer to increase the sample conductivity. The cross-section sample was prepared by soaking the membrane sample into liquid N_2_.

**Figure 3:**
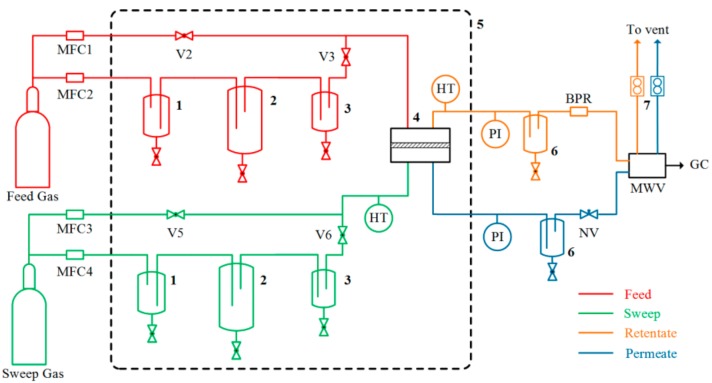
Mixed-gas permeation setup (**1**: MFC-safety trap; **2**: Humidifier; **3**: Droplets trap; **4**: Membrane module; **5**: Heated cabinet; **6**: Water knockout; **7**: Bubble flow meters; **MFC**: Mass flow controller; **NV**: Needle valve; **BPR**: Back-pressure regulator; **PI**: Pressure indicator; **HT**: Humidity and temperature sensor; **MWV**: Multi-way valve; **GC**: Gas chromatograph).

Gas-separation performance was measured in a humid mixed-gas permeation setup with adjustable RH, as schematically depicted in Figure 3. More details about the testing procedure can be found in our previous reports [35,36]. The RH value is controlled by using four mass flow controllers (El-Flow series, Bronkhorst, Veenendaal, Netherland). A CO_2_/N_2_ gas mixture (10/90 *v*/*v*) constituted the feed gas. CH_4_ was used as sweep gas instead of an inert gas (e.g., Helium) since Helium was used as the carrier gas for the gas chromatograph (GC). The feed pressure was set as 2.0 bar while the sweep side pressure was set to 1.05 bar. The composition of permeate stream was monitored by a calibrated gas chromatograph (490 Micro GC, Agilent, Santa Clara, CA, USA). For each permeation test, experiment lasted at least for 6 h until a steady state was reached.

The permeability coefficient (*P_i_*) of the *i*th penetrant species can be obtained by Equation (2):

(2) $$\;P_{m,i}=\frac{N_{perm}\left(1-y_{H_{2}O}\right)y_{i}l}{A\left(\langle p_{i,feed},\;p_{i,ret}\rangle -p_{i,perm}\right)}\;$$

where *N_perm_* is the total permeate flow measured with a bubble flow meter, *y*_*H*_2_*O*_ is the molar fraction of water in the permeate flow (calculated according to the RH value and the vapour pressure at the given temperature), *y_i_* is the molar fraction of the species of interest in the permeate and *p_i,feed_*, *p_i,ret_* and *p_i,perm_* identify the partial pressures of the *i*th species in the feed, retentate and permeate, respectively. It is worth mentioning that all the tests were repeated at least two times with the error lower than 5%, as such the error bars are not visible in the figures, thus not presented in the figures.

The separation factor is calculated by Equation (3):

(3) $$\alpha_{i/j}=\frac{y_{i}/x_{i}}{y_{j}/x_{j}}\;$$

## 3. Results and Discussion

### 3.1. Nanofiller Characterization

**Figure 4:**
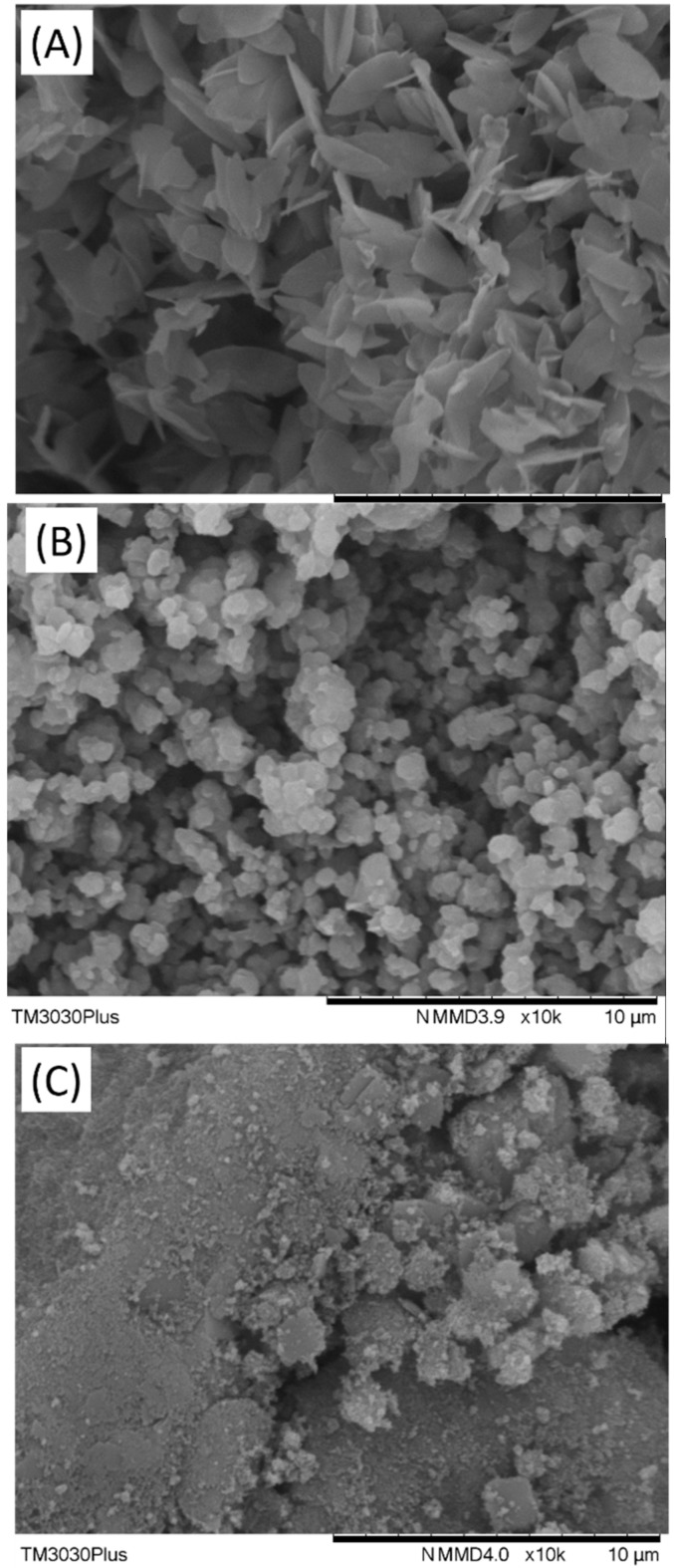
SEM image of the ZIF series used in the present study, (**A**) ZIF-L, (**B**)ZIF-8, and (**C**) ZIF-7.

The morphology of the four nanofillers used in the present study were investigated using SEM, results are shown in Figure 4.

As it can be seen in Figure 4, ZIF-L presents a leaf-sharp with a width of ~1.8 µm and length of ~4 µm, which is in good agreement with literature results [26]. The SEM image for ZIF-8 particles show obvious aggregation but overall particle size is still in the micrometre range. ZIF-7 was prepared based on the procedure reported in Ref. [23]. The SEM image of ZIF-7 also shows serious aggregation but the individual particles exhibit similar structure as literature [23]. It is worth mentioning that nanoparticles always tend to aggregate in a dry state but in this work the aggregations have been partly broken down to smaller particles via proper dispersion methods (e.g., stirring, ultra-sonication) and using proper solvent.

### 3.2. Thermal Properties

**Figure 5:**
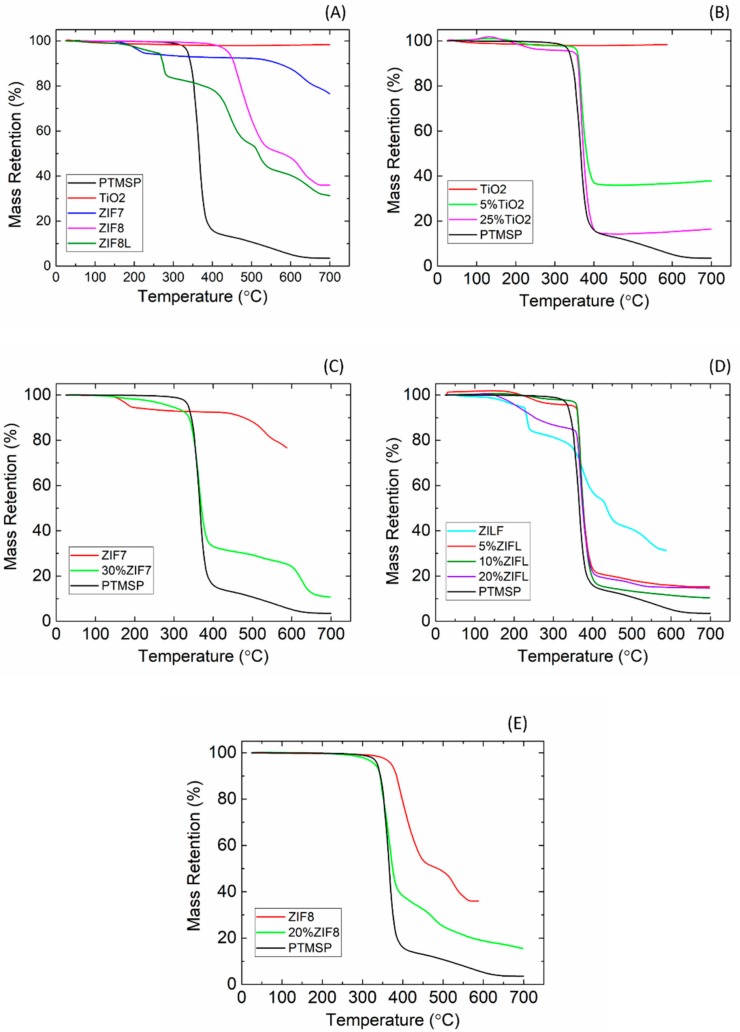
TGA results of neat PTMSP polymer and different nanoparticles (**A**), TGA curves of PTMSP/TiO_2_ (**B**), PTMSP/ZIF-7 (**C**), PTMSP/ZIF-L (**D**), and PTMSP/ZIF-8 (**E**).

Thermal stability of membranes is a property that is highly valued in membrane separation. TGA was used to study the influence of the nanofillers on the thermal stability of the hybrid membranes. The TGA results for PTMSP, ZIF-8, ZIF-L, ZIF-7 and TiO_2_ and relevant hybrid membranes are shown in Figure 5.

Among all the tested materials, the TiO_2_ nanoparticles present the best thermal stability. The weight-loss of TiO_2_ is less than 2 wt % of the original weight at 700 °C, showing that TiO_2_ is extremely stable in the given temperature range. A T_onset_ of 439 °C was obtained for the ZIF-8, which is in good agreement with the literature value [21]. At temperatures higher than the T_onset_, a steep reduction in weight is obtained, which is corresponding to the collapse of the ZIF-8 structure and carbonization under extreme thermal stress. A 66% weight-loss can be observed at 700 °C, which is also consistent with literature data [37]. Considering the ZIF-L, a weight loss of approximately 20% can be found at around 200 °C, which is due to the mass loss arising from the removal of the weakly linked ½ Hmim and guest water molecules [27]. Further increasing the temperature will result in decomposition of ZIF-L into zinc oxide [27]. In the case of ZIF-7 nanoparticles, a slight weight loss was observed at around 177 °C, which was a result of the evaporation of the DMF solvents (b.p. = 153 °C) from the particle cages [38]. Afterwards the sample mass kept stable until around 500 °C, where a second stage of weight loss was observed due to the decomposition of ZIF-7 into zinc oxide [38]. 

Figure 5B–E presents the TGA curves of the hybrid membranes with the four different types of nanofillers. In the case of TiO_2_ and ZIF-8, due to the superior thermal stability of the two nanofillers, adding them into PTMSP resulted in a hybrid membrane with comparable thermal stability to neat PTMSP but with higher residual mass at high temperatures. In the case of the ZIF-7 and ZIF-L, as these two nanofillers has relatively lower T_on-set_ compared to the PTMSP polymer, adding them into the polymeric phase slightly reduced the overall thermal stability of the hybrid membranes. However, no weight loss can be found below 150 °C, which fulfils the requirements for post-combustion CO_2_ capture process. 

### 3.3. Membrane Morphology

**Figure 6:**
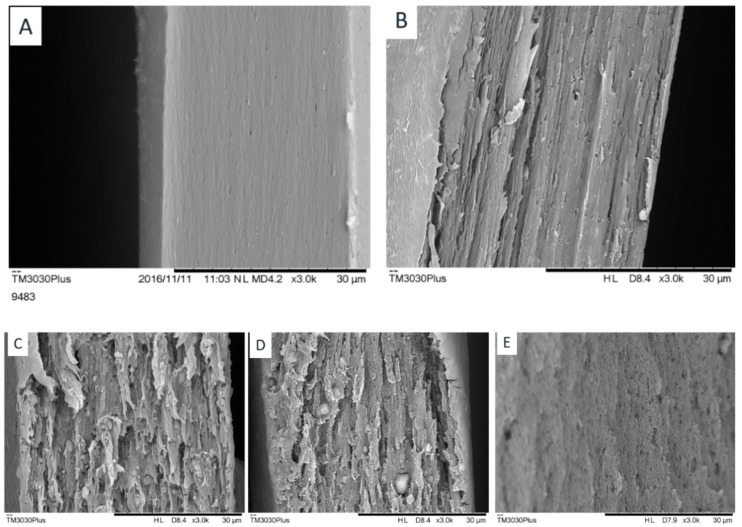
SEM images of membranes. (**A**) PTMSP, (**B**) PTMSP/30 wt % ZIF-7, (**C**) PTMSP/20 wt % ZIF-8, (**D**) PTMSP/20 wt % ZIF-L, and (**E**) PTMSP/20 wt % TiO_2_.

The membrane morphology is of critical importance for hybrid membranes. Depending on the affinity between the polymer and the fillers, hybrid membranes may be characterized by different polymer/particle interface and the transport properties of hybrid membranes are strongly dependent on the nanoscale morphology of the membranes [16]. Figure 6 display the cross section of the different membranes fabricated in the present study.

Figure 6A shows the SEM results for the neat PTMSP membrane, where a homogeneous morphology can be observed, as expected for a pristine polymeric phase. In the case of the PTMSP/ZIF-7 sample (Figure 6B), no obvious aggregation can be found from the membrane surface. However, from the cross-section image, the ZIF-7 nanoparticles seem to aggregate to one side of the membrane. In the case of the membrane with ZIF-8 and ZIF-L dispersed (Figure 6C,D), aggregation can be found in both membranes. The compatibility between the nanofillers and PTMSP seems not sufficient. In the TiO_2_ case, aggregates can be found on the membrane surface but no obvious aggregates are found from the cross-section images. Furthermore, different from other nanofillers, the TiO_2_ seems created additional voids or gas diffusion paths in the PTMSP membrane, which is expected to be beneficial for gas permeability but may be to an extent negative to the selectivity.

### 3.4. FTIR

**Figure 7:**
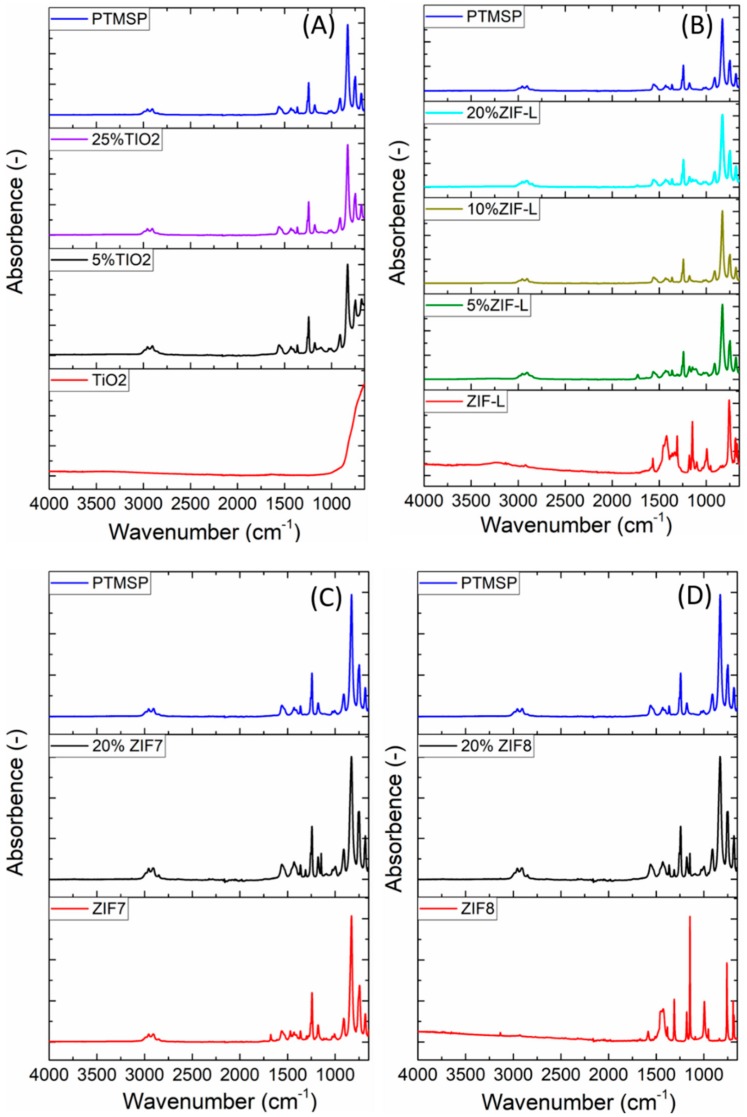
FT-IR spectrum of PTMSP/nanofiller hybrid membranes. (**A**) PTMSP/TiO_2_, (**B**) PTMSP/ZIF-L, (**C**) PTMSP/ZIF-7 and (**D**) PTMSP/ZIF-8.

FTIR was used to study the chemical structure of the nanofiller, PTMSP as well as the hybrid membranes. Results are shown in Figure 7. Detailed peak assignments are listed in Table 1.

Figure 7 depicts the FT-IR of pure PTMSP and different particles. Detailed peak assignments are also listed in Table 1. It is found that the neat PTMSP results are in accordance with the IR spectra given by Khodzhaeva et al. [39]. Three characteristic peaks can be found for PTMSP at 1540, 1240 and 820 cm^−1^, which are due to stretching of double C=C bond, deformation of Si–C–H bond and stretching of the Si–C bond. In the case of ZIF-7 sample. Two peaks can be observed at 1455 and 777 cm^−1^, corresponding to C–C bond and C–H bond stretching [23]. Considering ZIF-8 and ZIF-L samples, due to the similar chemical structure, identical peaks were obtained for these two samples. The characteristic peak for ZIF-8/ZIF-L locates at 1584 cm^−1^, which is due to the stretching of C–N bond found in the 2-methylimidazole ring [40]. The TiO_2_-particles have a rather non-distinct IR-spectrum, being approximately a line, until it gradually increases as it comes closer to 1000 cm^−1^. Murashkevich et al. [41] reported that the very broad peak will reach its maximum around 526 cm^−1^ and that there should be a “shoulder“ around 768 cm^−1^ due to symmetric stretching vibrations in the Ti–O bond. From Figure 7 it is observed that the TiO_2_ results matches with the literature value quite well.

The FTIR spectrum of the hybrid membrane with the four different nanofillers are also showing in Figure 7. Based on the results, no new peaks or peak position shift can be found, which denoting that no chemical interaction happens between the nanofillers and the polymeric matrix. In the case of TiO_2_, due to the fact that the FTIR spectrum of TiO_2_ is relatively simpler compared to PTMSP, the PTMSP spectrum is dominating in the hybrid membranes. For the other three additives, the peak intensity of the nanofillers changes in the hybrid membrane, which has good correlation with the content of the fillers in the hybrid membranes.

### 3.5. Mixed Gas Permeation Results

A CO_2_/N_2_ gas mixture (10/90 *v*/*v*) was used as feed gas to investigate gas separation properties of obtained hybrid membranes. The mixed gas permeation tests of all the studied membranes were carried out at room temperature with controlled RH level.

#### 3.5.1. Comparison of Two Different Solvents

It is well known that the solvents used to fabricate polymeric membranes can greatly affect the membrane properties [42] and that nanoparticles have different dispersion properties in different organic solvents [43]. In the present study, we found that ZIF-7 had relatively better dispersion property in chloroform while ZIF-8, ZIF-L and TiO_2_ could be dispersed better in cyclohexane, thus both solvents were employed in fabrication of hybrid membrane. 

**Figure 8:**
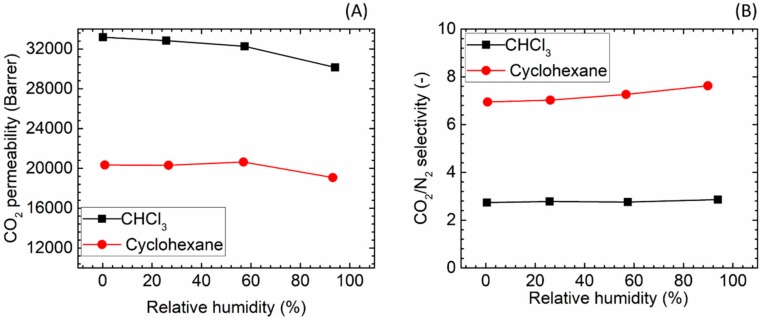
Gas separation performances of PTMSP membrane prepared from two different solvents, (**A**) CO_2_ permeability and (**B**) CO_2_/N_2_ selectivity.

CO_2_/N_2_ separation performances of the two neat PTMSP membranes prepared using chloroform and cyclohexane solvents were firstly studied as the blank data for the comparison with the hybrid membranes. The permeation results are shown in Figure 8. The lower boiling point (61 °C) of chloroform led to a higher CO_2_ permeability (33,169 bar) compared to the one observed for cyclohexane (b.p. 81 °C) (20,338 bar), even though a lower CO_2_/N_2_ separation factor was obtained (2.7 for chloroform versus 6.9 for cyclohexane, Figure 8B). The results achieved for both solvents are similar to literature values [44,45,46]. The different solvent volatility is believed has led to different rearrangement of the polymeric chain in the final matrix: in the case of the less volatile solvent, the longer time needed to obtain the solid film may result in a higher polymer chain packing density.

Figure 8 also presents the effect of the relative humidity level in the feed gas. As can be seen, the CO_2_ separation performances appear only to a small extent being affected by an increase of the humidity content in the gaseous stream, as both membranes showing limited change in CO_2_ permeability (~10% drop) followed by a slight increase (~10%) in CO_2_/N_2_ selectivity. Interestingly, literatures on hydrophobic polymers [47,48] showed that water vapour significantly affects the transport of incondensable gases through the polymer matrix, despite of the limited H_2_O uptake to the membrane. However, the strong effect of humidity is not observed for PTMSP, most likely due to that the different transport mechanism; in the PTMSP-based membranes, separation is related to the free volume size and distribution within the polymeric matrix. Scholes et al. [49] also reported a limited effect of water vapour on the CO_2_ permeability of PTMSP casted using toluene as solvent, which suggests that the negligible effect of water vapour is independent of the type of solvent used during the membrane fabrication procedure.

#### 3.5.2. PTMSP/ZIF-8 Hybrid Membranes

In the case of ZIF-8 hybrid membrane, 20 wt % ZIF-8 was added into PTMSP matrix. The amount of the ZIF-8 was determined based on literature values. 

**Figure 9:**
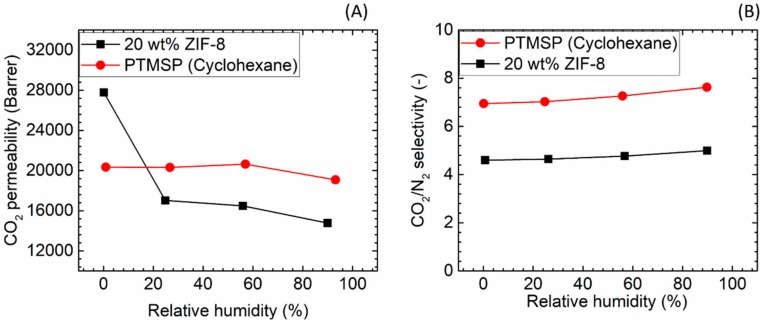
Gas separation performance of PTMSP/ZIF-8 hybrid membranes. (**A**) CO_2_ permeability and (**B**) CO_2_/N_2_ selectivity.

As shown in Figure 9, at dry state, adding ZIF-8 into PTMSP results in a sharp increase in CO_2_ permeability (27,781 bar, +37%) with a decrease of CO_2_/N_2_ selectivity (4.5, −36%) compared to the neat PTMSP membrane. The higher permeability and lower selectivity at dry state can be attributed to the micro-voids between the nanofillers and polymer phase, which is observed also from the SEM images (Figure 6C). Similar results have also been achieved for membranes with ZIF-8 embedded in PIM-1 [50], in which a comparable loading of ZIF-8 (28 wt %) showed a trend similar to the one observed in this study for both CO_2_ permeability and CO_2_/N_2_ selectivity (the CO_2_ permeability was tuned to values in the same range of PTMSP by ethanol swelling). 

From Figure 9B it can be seen that with a small amount of water vapour in the feed gas (RH ≈ 25%), the CO_2_ permeability dramatically decreased to 17,029 bar, reaching a performance that is 15% lower than that of the neat PTMSP membranes at the same RH condition (20,315 bar). Water adsorption in ZIF-8 was reported to be relatively low compare to other polar molecules [51]. However, the imidazolium linker introduces a –NH functionality on the outer surface of the ZIF cage, causing the H_2_O molecules to cover preferentially the crystal structure rather than to be adsorbed in the cage [51]. This effect may reduce the free volume at the polymer/particles interface, negatively affecting the gas transport through the membrane. Experiments show that further increase of the RH value causes a limited drop in CO_2_ permeability for the PTMSP/ZIF-8 hybrid membranes. Surprisingly, the humidity level had a very limited effect on the selectivity of the hybrid membrane, suggesting that the gas transport is mainly through the polymeric matrix and at the polymer/particles interface, not through the pores of the fillers, which should give a much higher selectivity.

#### 3.5.3. PTMSP/ZIF-L Hybrid Membranes

In order to fully exploit the sieving ability of the 2D morphology of nanofillers, the assessment of the influence of porous 2D nanosheets in membranes has attracted great attention in recent years [52,53,54,55]. However, little effort has been dedicated to ZIF-based porous nanosheets, or in particular, not in gas separation membranes. To the best of our knowledge, only one report was found using ZIF-L as nanofiller in hybrid membranes for gas separation [28].

**Figure 10:**
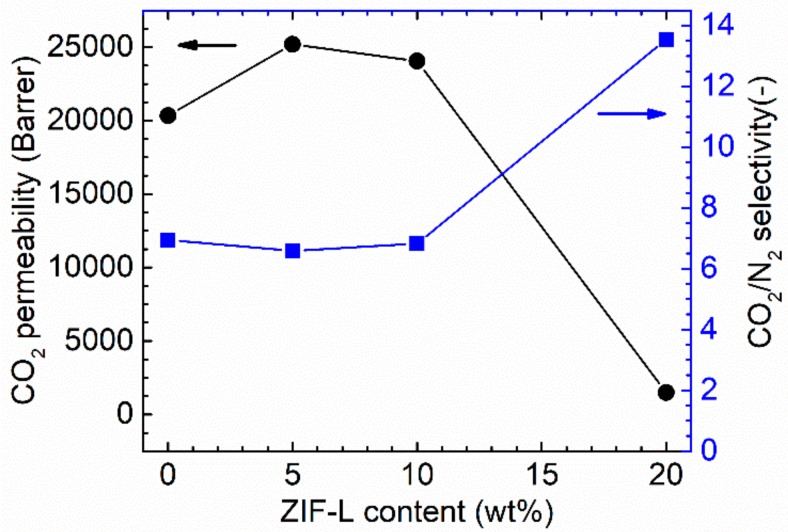
Gas separation performances of PTMSP/ZIF-L hybrid membranes as a function of the ZIF-L loading.

**Figure 11:**
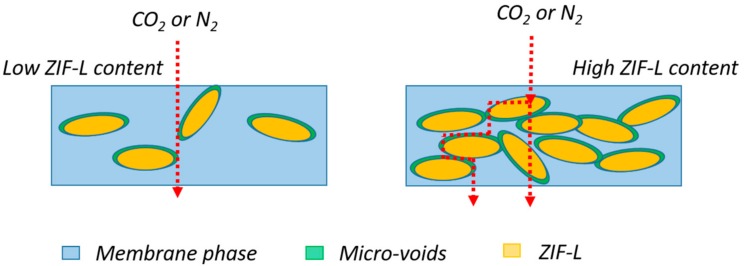
Possible gas transport mechanism in PTMSP/ZIF-L membranes.

In this work, we investigated the effect of ZIF-L loadings in PTMSP membrane by preparing samples containing three different ZIF-L loadings (5 wt %, 10 wt % and 20 wt %). As can be seen in Figure 10, adding 5 wt % and 10 wt % ZIF-L into PTMSP matrix significantly improves CO_2_ permeability and the 5 wt % loading seems the most effective. Surprisingly, a further increase of the ZIF-L content to 20 wt % causes a dramatic decrease in CO_2_ permeability (1489 bar), which is one order of magnitude lower than the neat PTMSP value. The opposite trend is observed for the CO_2_/N_2_ selectivity: at a ZIF-L content of 5 wt % and 10 wt %, the CO_2_/N_2_ selectivity value is almost unchanged. When 20 wt % ZIF-L is presented in the PTMSP matrix, the CO_2_/N_2_ selectivity increases from 7 to 13~14. A similar behaviour (initial permeability increase followed by significant decrease) is also reported for other porous 2D nanosheets [53,55]. A graphic representation of the possible gas transport mechanism is proposed and shown in Figure 11. Compared to PTMSP membranes, membranes made of neat ZIF-L particles have relatively lower CO_2_ permeability [26]. At lower loadings, increased gas permeability mainly comes from the micro-voids between the ZIF-L and PTMSP. As the addition of ZIF-L introduces more less permeable regions in the PTMSP matrix, which increases the gas diffuse path way in the membrane and part of the gases diffuse through the ZIF-L phase, leading to much lower permeability and relatively higher CO_2_/N_2_ selectivity. It has been reported that the neat ZIF-8 or ZIF-L membranes normally show relatively low CO_2_/N_2_ selectivity (in the range of <5) [56,57]. However, according to literature, when the ZIF-8 nanoparticles were added into polymer matrix, gas selectivity of the hybrid membranes are significantly higher [20,21]. Therefore, the intrinsic selectivity of the ZIF-8 material is possibly higher than the selectivity obtained from the ZIF membranes, in which the defects and micro voids between the ZIF-8 crystals may reduce the overall selectivity.

**Figure 12:**
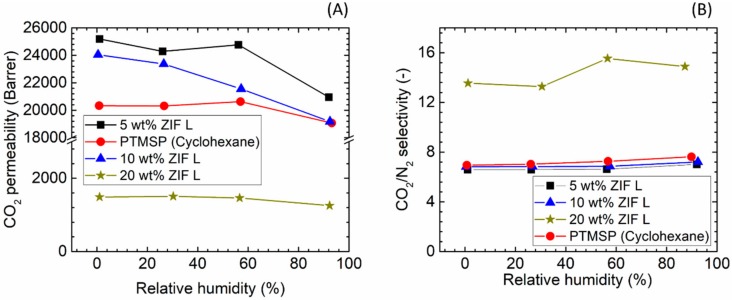
Gas separation performances of PTMSP/ZIF-L hybrid membranes as a function of relative humidity in the gaseous stream. (**A**) CO_2_ permeability and (**B**) CO_2_/N_2_ selectivity.

The influence of water vapour on the separation performance of the ZIF-L-based hybrid membranes is shown in Figure 12. Increasing the water vapour content in the gaseous streams resulted in a decrease in CO_2_ permeability for all the membranes containing ZIF-L fillers. For the 5 and 10 wt % loading the drop is limited to 17–20% of the dry value and the CO_2_ transport remains above the value of the neat PTMSP membranes. In the case of 20 wt % ZIF-L loading, a similar decrease (15.7%) was observed. In terms of the neat PTMSP membrane, the CO_2_ permeability decreased only 6% with the RH level increasing from 0% RH to 100% RH. Similar to the ZIF-8 case, the presence of water vapour shows a negligible effect on the CO_2_/N_2_ selectivity, independently from the particles loading within the membrane matrix.

#### 3.5.4. PTMSP/ZIF-7 Hybrid Membranes

**Figure 13:**
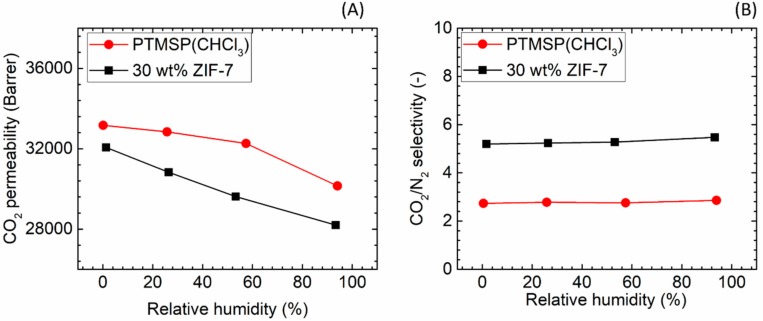
Gas separation performances of PTMSP/ZIF-7 hybrid membranes. (**A**) CO_2_ permeability and (**B**) CO_2_/N_2_ selectivity.

Based on literature, a higher ZIF-7 loading in a hybrid membrane can be beneficial to improving CO_2_ selectivity over other gases (e.g., N_2_ and/or CH_4_). According to Li et al., the highest selectivity was obtained at a ZIF-7 content of 34 wt % [23]. In another report, the highest CO_2_/CH_4_ selectivity was obtained at a ZIF-7 content of 35 wt % [24]. Therefore, in this study, 30 wt % ZIF-7 nanoparticles were determined as the optimal loading in the PTMSP/ZIF-7 hybrid membrane. The gas permeation results of the resultant hybrid membranes are shown in Figure 13.

As indicated in Figure 13, even with the ZIF-7 loading of up to 30 wt % in the hybrid membrane, only a minor change in the CO_2_ permeability (from 33,169 bar to 32,065 bar) was observed. Similar results have been reported in Pebax/ZIF-7 membranes, in which a ZIF-7 content of 22~34 wt % resulted in a reduced CO_2_ permeability and much improved CO_2_/N_2_ selectivity [23,24,25]. In another study, ZIF-7 particles were added into the polybenzimidazole (PBI) matrix to improve CO_2_/H_2_ separation performances. According to their results, adding 50 wt % of ZIF-7 nanoparticles into PBI membranes only increased CO_2_ permeability from 0.4 to 1.8 Bar, indicating that the intrinsic ZIF-7 permeability is in the low region [58]. Nevertheless, adding ZIF-7 nanoparticles into Pebax and PBI has been reported to be rather efficient in promoting CO_2_ selectivity over other gases [23,25]. In this work, the CO_2_/N_2_ selectivity was doubled by adding 30 wt % of ZIF-7 into PTMSP matrix (from 2.7 to 5.2). Although the absolute value is still too low, it is a significant improvement compared to the neat PTMSP membranes. 

#### 3.5.5. PTMSP/TiO_2_ Hybrid Membranes

**Figure 14:**
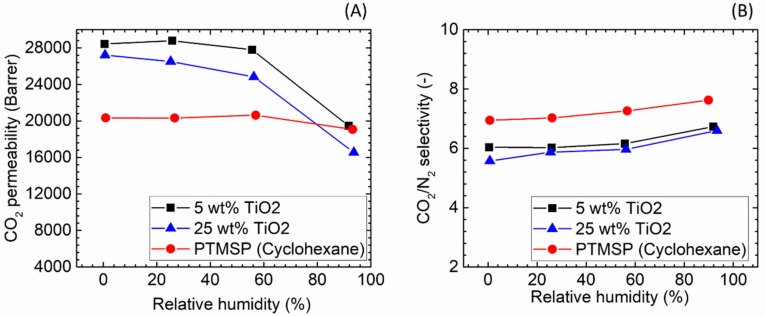
Gas separation performance of PTMSP/TiO_2_ hybrid membranes (Cyclohexane used as solvent). (**A**) CO_2_ permeability and (**B**) CO_2_/N_2_ selectivity.

Compared to the ZIF series, the addition of TiO_2_ nanoparticles showed quite different effects. At dry state, adding 5 wt % TiO_2_ nanoparticles into PTMSP matrix significantly improved the CO_2_ permeability (28,432 bar, 40% increased) but the increase in TiO_2_ content (25 wt %) did not show further increase in the permeability (Figure 14). It is frequently reported that the increase of CO_2_ permeability in hybrid membranes containing TiO_2_ is related mainly to the formation of interfacial voids at the polymer/particles interface and to the disruption of polymer chain packing induced by the nanoparticles rather than to its high CO_2_ adsorption capacity [5]. This observation is also confirmed in this study by the reduction of the CO_2_/N_2_ selectivity by the addition of TiO_2_ nanofillers in PTMSP hybrid membranes.

Interestingly, the presence of water vapour in the feed gas reduced the gas permeability to a significant extent. It was found that, when increasing the RH value from 75% to 100%, both membranes with 5 and 25 wt % TiO_2_ nanoparticles exhibited a notable decrease (35–40%) in the CO_2_ permeability, which may be due to the competitive sorption of water vapour that occupies the additional free volume created by the TiO_2_ nanoparticles. Similar to all the cases in this work, the presence of water vapour showed a negligible effect on the selective feature of the hybrid membranes.

The gas separation performances of the hybrid membranes various additives are listed in Table 2.

As can be seen from Table 2, adding ZIF-8 and TiO_2_ into PTMSP improves CO_2_ permeability but the improvement is at the expenses of the CO_2_/N_2_ selectivity. Adding ZIF-7 into PTMSP leads to lower CO_2_ permeability and slightly higher CO_2_/N_2_ selectivity. In the case of ZIF-L, at lower particles content, higher CO_2_ permeability and lower CO_2_/N_2_ selectivity was observed but a further increase of the ZIF-L loading causes a significant improvement of the CO_2_/N_2_ selective feature but with a dramatic drop in the gas permeability coefficient. In a similar fashion for all the investigated nanoparticles, the presence of water vapour negatively affects the CO_2_ permeability of the hybrid membranes, with the ZIF-8 and the TiO_2_ based membranes being the most affected. No significant effect is, however, observed for the selective feature of the hybrid membranes. 

## 4. Conclusions

In the present study, a broad range of fillers were used for the fabrication of hybrid membranes for CO_2_ capture. Three different ZIF-type porous nanofillers (2D and 3D) and TiO_2_ nanoparticles (0D) were utilized to fabricate PTMSP-based hybrid membranes, aiming at the improvement of the CO_2_ separation performance. The TGA analysis shows that the hybrid membranes are characterized by high thermal stability compared to the pristine polymer, fulfilling the requirements for most CO_2_ separation applications. In addition, the SEM images indicates that the particles are homogeneously distributed within the polymer matrix but the poor affinity with the polymer phase caused the formation of interfacial voids.

The solvent used for the membrane preparation was found very important: the CO_2_ permeability decreased by 40% while the selectivity increased by 150% simply by changing the solvent in the membrane casting solution from chloroform to cyclohexane. The loadings of the different nanofillers makes big differences in the separation property enhancement. Adding ZIF-8 and TiO_2_ particles into PTMSP matrix has always led to an increment in CO_2_ permeability. On the other hand, the addition of ZIF-7 into the hybrid membranes resulted in a relatively lower CO_2_ permeability but higher CO_2_/N_2_ selectivity. The most notable variation was obtained from the addition of ZIF-L, the porous 2D nanosheets. At a low ZIF-L loading, the CO_2_ permeability was enhanced but further increase of the particle loading caused in a dramatic decrease in CO_2_ permeability but doubled CO_2_/N_2_ selectivity compared to the neat PTMSP membrane. For all the hybrid membranes, increasing the water vapour content in the gaseous streams has caused a reduction in CO_2_ permeability with limited effect on the CO_2_/N_2_ selectivity. 

To sum up, despite the different morphology and properties of the selected nanofillers in this study (porous/non-porous, 0 to 3D nanostructures), adding the nanofillers into PTMSP either leads to higher permeability with lower CO_2_/N_2_ selectivity, or vice versa. 

## Figures and Tables

**Table 1:** Detailed peak assignment of PTMSP and various additives.

	Peak Position (cm^−1^)	Peak Assignment	Ref.
PTMSP	1540	stretching of the double C=C bond	[39]
	1240	deformation of the SiC–H bond	
	820	stretching of the C–Si bond	
ZIF7	1455	C–C stretching	[23]
	777	C–H stretching	
ZIF8/ZIFL	1584	stretching of C–N bond found in the 2-methylimidazole ring	[40]
	1350–1500	ring stretching	
	900–1350	coupled with in-plane ring bending	
	800	out-of-plane bending	
	1146	C–H vibrations	
	1310	C–H vibrations	
TiO_2_	768	symmetric stretching vibrations in the Ti–O bond	[41]

**Table 2:** Summary of PTMSP hybrid membrane separation performances.

Solvent	Nanofiller	Nanofiller Content (wt %)	RH (%)	CO_2_ Permeability	CO_2_/N_2_ Selectivity (-)
CHCl_3_	-	-	0.2	33,169.3	2.7
CHCl_3_	-	-	94.1	30,152.0	2.9
CHCl_3_	ZIF-7	30	1.3	32,065.0	5.2
CHCl_3_	ZIF-7	30	93.4	28,205.3	5.5
Cyclohexane	-	-	0.9	20,338.7	6.9
Cyclohexane	-	-	93.2	19,074.8	7.6
Cyclohexane	TiO_2_	5	0.5	28,432.2	6.0
Cyclohexane	TiO_2_	5	91.7	19,465.6	6.7
Cyclohexane	TiO_2_	25	0.7	27,222.0	5.6
Cyclohexane	TiO_2_	25	93.6	16,550.1	6.6
Cyclohexane	ZIF-8	20	0.7	27,781.7	4.6
Cyclohexane	ZIF-8	20	89.9	14,764.1	5.0
Cyclohexane	ZIF-L	5	1.1	25,191.4	6.6
Cyclohexane	ZIF-L	5	92.0	20,949.6	7.0
Cyclohexane	ZIF-L	10	0.5	24,046.1	6.8
Cyclohexane	ZIF-L	10	92.5	19,175.1	7.2
Cyclohexane	ZIF-L	20	1.1	1,489.2	13.5
Cyclohexane	ZIF-L	20	92.3	1,255.1	14.9
